# Supplementary material for: Deletion of wfs1 Impairs Oligodendrocyte Precursor Cells Dorsal Distribution and Myelination Through the wfs1-hmgcs1 Axis in Zebrafish
Source: Biology (Basel). 2026 Aug 16;15(16):1402. doi: 10.3390/biology15161402 (PMC13510151; doi:10.3390/biology15161402)
Supplement: Supplementary file 1 [file biology-15-01402-s001.zip › File S1-Transcriptome sequencing data and differentially expressed genes (DEGs) in wfs1-- zebrafish at 5 dpf/2.KEGG/wfs1KOvsWT/UP/src/dre00603.html]

dre00603


- dre:450083

- Up regulated genes

450083(2.11033262833863)

- dre:447881

- Up regulated genes

447881(2.48260563326135)
- dre:100005375

- Up regulated genes

100005375(2.4224086269223)

- dre:447881

- Up regulated genes

447881(2.48260563326135)
- dre:100005375

- Up regulated genes

100005375(2.4224086269223)

- dre:447881

- Up regulated genes

447881(2.48260563326135)
- dre:100005375

- Up regulated genes

100005375(2.4224086269223)

- dre:447881

- Up regulated genes

447881(2.48260563326135)
- dre:100005375

- Up regulated genes

100005375(2.4224086269223)

Close
